# A Bayesian analysis of finerenone in heart failure with mildly reduced and preserved ejection fraction: a pre-specified analysis of FINEARTS-HF

**DOI:** 10.1093/ehjcvp/pvag010

**Published:** 2026-02-16

**Authors:** Alasdair D Henderson, Kieran F Docherty, Atefeh Talebi, Toru Kondo, Mark C Petrie, Brian L Claggett, Akshay S Desai, Muthiah Vaduganathan, John J Atherton, Johan Bauersachs, Morten Schou, Subodh Verma, Carolyn S P Lam, Bertram Pitt, Michele Senni, Sanjiv J Shah, Adriaan A Voors, Faiez Zannad, Meike Brinker, Flaviana Amarante, Katja Rohwedder, James Lay-Flurrie, Scott D Solomon, John J V McMurray, Pardeep S Jhund

**Affiliations:** BHF Glasgow Cardiovascular Research Centre, School of Cardiovascular and Metabolic Health, University of Glasgow, Glasgow G12 8TA, UK; BHF Glasgow Cardiovascular Research Centre, School of Cardiovascular and Metabolic Health, University of Glasgow, Glasgow G12 8TA, UK; BHF Glasgow Cardiovascular Research Centre, School of Cardiovascular and Metabolic Health, University of Glasgow, Glasgow G12 8TA, UK; BHF Glasgow Cardiovascular Research Centre, School of Cardiovascular and Metabolic Health, University of Glasgow, Glasgow G12 8TA, UK; Department of Cardiology, Nagoya University Graduate School of Medicine, Nagoya 466-8550, Japan; BHF Glasgow Cardiovascular Research Centre, School of Cardiovascular and Metabolic Health, University of Glasgow, Glasgow G12 8TA, UK; Cardiovascular Division, Brigham and Women’s Hospital, Harvard Medical School, Boston, MA 02115, USA; Cardiovascular Division, Brigham and Women’s Hospital, Harvard Medical School, Boston, MA 02115, USA; Cardiovascular Division, Brigham and Women’s Hospital, Harvard Medical School, Boston, MA 02115, USA; Department of Cardiology, Royal Brisbane and Women’s Hospital, University of Queensland Faculty of Medicine, Brisbane 4029, Australia; Department of Cardiology and Angiology, Hannover Medical School, Hannover 30625, Germany; Department of Cardiology, Herlev-Gentofte University Hospital, Hellerup 2900, Denmark; Division of Cardiac Surgery, St. Michael’s Hospital, Toronto M5B 1W8, Canada; National Heart Centre Singapore & Duke-National University of Singapore, Singapore 169609, Singapore; School of Medicine, University of Michigan, Ann Arbor, MI 48109, USA; School of Medicine & Surgery, University Bicocca Milan, Milan 20126, Italy; Cardiology Unit, Papa Giovanni XXIII Hospital, Bergamo 24127, Italy; Division of Cardiology, Northwestern University Feinberg School of Medicine, Chicago, IL 60611, USA; Department of Cardiology, University of Groningen, Groningen 9713 GZ, Netherlands; Inserm Clinical Investigation Centre, CHU, Université de Lorraine, Nancy 54500, France; Research & Development, Pharmaceuticals, Bayer AG, Wuppertal 42096, Germany; Cardiology and Nephrology Clinical Development, Bayer SA, São Paulo 04779-900, Brazil; Global Medical Affairs, Berlin 13353, Germany; Research & Development, Pharmaceuticals, Bayer plc, Reading RG2 6AD, UK; Cardiovascular Division, Brigham and Women’s Hospital, Harvard Medical School, Boston, MA 02115, USA; BHF Glasgow Cardiovascular Research Centre, School of Cardiovascular and Metabolic Health, University of Glasgow, Glasgow G12 8TA, UK; BHF Glasgow Cardiovascular Research Centre, School of Cardiovascular and Metabolic Health, University of Glasgow, Glasgow G12 8TA, UK

**Keywords:** Finerenone, Mineralocorticoid receptor antagonist, Heart failure with preserved ejection fraction, Bayesian

## Abstract

**Aims:**

The non-steroidal mineralocorticoid receptor agonist (MRA) finerenone reduced heart failure events and cardiovascular death in patients with heart failure with mildly reduced or preserved ejection fraction (HFmrEF/HFpEF) in a frequentist analysis of the FINEARTS-HF trial. We applied Bayesian methods including prior information to provide probabilistic estimates of efficacy and safety. The aim of this study was to estimate the probability of different magnitudes of treatment benefit with finerenone vs. placebo using Bayesian analysis of FINEARTS-HF.

**Methods and results:**

In this pre-specified Bayesian analysis, we estimated treatment efficacy incorporating prior information in a robust meta-analytic predictive (MAP) prior using data from two finerenone trials (FIDELIO-DKD and FIGARO-DKD) and a steroidal MRA spironolactone (TOPCAT). We compared results using vague priors and informative MAP priors. The primary outcome was cardiovascular death and total heart failure events. Among 6001 patients, Bayesian analysis with vague priors confirmed the frequentist results of a reduction in the rate of the primary outcome [RR 0.83; 95% credible interval (CrI) 0.74–0.94]. Incorporating prior evidence increased the posterior probability of a ≥ 10% reduction in the primary event rate from 90% to 92%. The probability that finerenone reduced cardiovascular death was 80% (HR 0.93, 95% CrI: 0.79–1.10), and all-cause mortality was 85% (HR 0.94, 95% CrI: 0.83–1.06). Finerenone increased the probability of hyperkalaemia and decreased the probability of hypokalaemia.

**Conclusion:**

The non-steroidal MRA finerenone reduced the rate of cardiovascular death and total heart failure events under both frequentist and Bayesian inference methods. The probability of benefit exceeded 80% for both cardiovascular and all-cause mortality with finerenone.

**Trial Registration**: ClinicalTrials.gov Identifier: NCT04435626

## Introduction

Randomized clinical trials are typically analysed using a frequentist approach where the aim is to use the evidence gathered to reject the null hypothesis, i.e. the hypothesis that there is no meaningful difference between the treatment groups. Under this framework, unobservable and fixed parameters of interest from statistical models such as the treatment effect (e.g. hazard ratios from a Cox proportional hazards model) are estimated. We subsequently estimate the probability that we would observe a treatment effect as (or more) extreme than the one observed if there was no difference between treatments and accept <5% as a ‘significant’ result.

Bayesian methods offer an alternative approach,^1-3^ where the treatment effect is a random variable to be estimated so we can make probability statements about the treatment effect. As recently recognized by the Food and Drug Administration (FDA),^4^ Bayesian analyses also offer the opportunity to formally incorporate prior information from previous studies. Bayesian analysis can update prior knowledge with new data through application of Bayes theorem. New data can update prior beliefs to provide a new estimate on a research topic which considers the totality of evidence rather than analysing each new study in isolation. Finally, the analyses can be used to support the primary inference in the trial.

The steroidal mineralocorticoid receptor antagonist (MRA) spironolactone was tested in patients with heart failure with mildly reduced or preserved ejection fraction (HFmrEF/HFpEF) in the TOPCAT trial (Treatment of Preserved Cardiac Function Heart Failure with an Aldosterone Antagonist).^5^ Spironolactone did not reduce the primary endpoint, and further analysis of the trial suggested that patients enrolled in two countries may not have had heart failure (HF) and did not take randomized treatment.^6,7^ After excluding these countries *post hoc*, there was a nominally significant effect of spironolactone on the primary outcome.

Whether or not MRAs might benefit patients with HFmrEF/HFpEF was tested in a recent trial with the non-steroidal MRA, finerenone. The FINEARTS-HF trial (FINerenone trial to investigate Efficacy and sAfety superioR to placebo in paTientS with Heart Failure) was designed and analysed using a frequentist approach.^8,9^ Finerenone significantly reduced the composite primary outcome of cardiovascular death and total HF events (rate ratio 0.84; 95% confidence interval, 0.74 to 0.95; *p* = 0.007). This analysis did not incorporate prior knowledge of the effect of MRAs in HFmrEF/HFpEF from TOPCAT, or the effect of finerenone in a population with chronic kidney disease and type 2 diabetes (FIDELIO-DKD and FIGARO-DKD; Finerenone in Reducing Kidney Failure and Disease Progression in Diabetic Kidney Disease and Finerenone in Reducing Cardiovascular Mortality and Morbidity in Diabetic Kidney Disease).^10,11^

We have analysed the FINEARTS-HF trial using Bayesian methods to incorporate prior knowledge from these trials with spironolactone and finerenone to estimate the probability of various treatment effect sizes and summarize relative and absolute treatment effects.

## Methods

### The FINEARTS-HF trial

FINEARTS-HF (NCT04435626) was a prospective, randomized, double-blind, placebo-controlled, trial which examined the efficacy and safety of finerenone compared with placebo, in patients with HFmrEF/HFpEF. The design, baseline characteristics, and primary results are published.^8,9,12^ Ethics Committees for the participating institutions approved the protocol and all patients gave written consent. Briefly, key inclusion criteria were age >40 years, symptomatic HF in New York Heart Association (NYHA) functional class II–IV, treatment with a diuretic for >30 days before randomization, and a left ventricular ejection fraction (LVEF) ≥ 40% with evidence of structural heart disease (either left atrial enlargement or left ventricular hypertrophy) measured within 12 months of screening. Patients were also required to have elevated natriuretic peptide levels (NT-proBNP >300 pg/mL [or BNP >100 pg/mL] for patients in sinus rhythm or NT-proBNP >900 pg/mL [or BNP >300 pg/mL] for patients in atrial fibrillation), measured within 90 days in those with a recent worsening HF event within 90 days of randomization, or measured 30 days before randomization in those without a recent worsening HF event. Both ambulatory and hospitalized patients were eligible for enrolment. Patients with prior LVEF <40% with subsequent improvement to ≥40% were also eligible for enrolment provided that ongoing HF symptoms were present and all other inclusion criteria were satisfied. Key exclusion criteria were eGFR <25 mL/min/1.73 m2, serum/plasma potassium >5.0 mmol/L at screening or randomization, or symptomatic hypotension with mean systolic blood pressure <90 mmHg at screening or randomization. The primary trial outcome was the composite of cardiovascular death and total (first and recurrent) HF events (i.e. HF hospitalization or urgent HF visit). Prespecified secondary outcomes were cardiovascular death, the total number of HF events; improvement in NYHA class from baseline to 12 months; change in the Kansas City Cardiomyopathy Questionnaire (KCCQ) total symptom score (KCCQ-TSS) from baseline to 6, 9, and 12 months; a composite kidney endpoint (defined as a sustained decrease in eGFR ≥50% relative to baseline over at least 4 weeks, or sustained eGFR decline <15 mL/min/1.73 m^2^, or initiation of dialysis or renal transplantation); and all-cause death. All deaths and potential primary outcome non-fatal events were adjudicated by an independent, blinded, committee. Prespecified safety analyses included serious adverse events, investigator-reported hyperkalaemia and changes in laboratory parameters of interest (e.g. elevations or decreases in potassium, elevations in serum creatinine, and decreases in systolic blood pressure).

### Bayesian priors for FINEARTS-HF

To allow a more direct comparison with the primary frequentist result we used a *vague* prior. A vague prior provides negligible prior knowledge about the expected treatment effect prior to the study and therefore conclusions are defined by the newly observed data, analogous to the frequentist framework.

A second set of analyses used *informative* priors estimated from previously reported clinical trials in the field. We used three sources of data to derive prior distributions for the efficacy of finerenone: (1) a previous study of a steroidal MRA in patients with HFpEF (TOPCAT), (2) TOPCAT data from the Americas region only because of previously reported regional variation in TOPCAT results,^6^ and (3) the prespecified FIDELITY pooling of FIDELIO-DKD and FIGARO-DKD (FInerenone in chronic kidney diseasE and type 2 diabetes: Combined FIDELIO-DKD and FIGARO-DKD Trial programme analysis)^13,14^ (*Table 1*). We used these results separately to define prior distributions from the reported log rate ratios, downweighed by half (by doubling the standard deviations). We defined a fourth prior which was a composite of TOPCAT (Americas region only) and FIDELITY results, and constructed using the robust meta-analytic predictive (MAP) prior method.^16^ This meta-analysis of the log rate ratio estimates of the previously described trials are summarized in a single distribution including a component which downweighs the prior by an arbitrary 10%^16^ (see Supplementary material online, *Figure S1*). This method allows for prior information to be incorporated from multiple studies at once without inflating the influence of the prior over the posterior distribution or selectively down weighting particular prior information because of differences with the newly collected data. We performed a further sensitivity analysis by using all TOPCAT data in the MAP prior estimation. We used the same methodology to derive a specific MAP prior for cardiovascular death and all-cause death using previous estimates of the log hazard ratio from these two sources.^6,14^

**Table 1:** Definition of prior distributions for the rate ratio of treatment for total heart failure hospitalizations and cardiovascular death

Prior	Population	Frequentist estimate	Prior distribution (log rate ratio)	Prior probability of treatment benefit (RR < 1.0)
Vague	—	—	∼N(0, 0.5)	50%
TOPCAT^15^	HFmrEF/HFpEF	0.84 (95% CI: 0.70–1.02)	∼N(−0.174, 0.19)^a^	82%
TOPCAT-Americas^15^	HFmrEF/HFpEF	0.79 (95% CI: 0.64–0.97)	∼N(−0.236, 0.21)^a^	87%
FIDELITY^13^	Type II diabetes & chronic kidney disease	0.82 (95% CI: 0.72–0.95)	∼N(−0.198, 0.10)^a^	92%
Meta Analytic Predictive^16^	Composite	—	∼N(−0.188, 0.20)	81%

^a^Standard deviations are doubled from the published frequentist estimates to partially account for uncertainty between previous findings and new data.

### Bayesian analysis of FINEARTS-HF

Our Bayesian analysis of FINEARTS-HF updates collective *prior* knowledge about the treatment effect with observed data from the trial through a defined *likelihood* function. Using simulation-based methods to combine the prior and current information we derived an updated *posterior* distribution of treatment efficacy. We followed reporting guidelines of Bayesian analyses (see Supplementary material online, *Table S1*).^17-19^

### Statistical analysis

We analysed the same population as the primary frequentist analysis of FINEARTS-HF. The primary endpoint of cardiovascular death and total (first and recurrent) HF events and was analysed using a stratified Andersen-Gill proportional hazards model with clustered standard errors by participant (LWYY) with strata defined by randomization strata [region and LVEF (< 60%, ≥60%)]. We used a vague prior as our primary analysis to compare with the frequentist results, then used the four prior distributions described earlier (*Table 1*), each of which resulted in a posterior distribution of treatment efficacy to allow comparison of the impact of different informative priors. To measure treatment effects on an absolute scale we used a negative binomial likelihood, adjusted for randomization strata and with an offset term for follow-up time. We used these models to estimate treatment-specific predictions of the rates and their difference per 100 person-years.

For secondary efficacy endpoints all time-to-first-event secondary endpoints were analysed using a stratified Cox regression likelihood with the same strata as above. Total HF events were analysed with the same LWYY as the primary composite outcome. Change in NYHA was modelled with a binomial likelihood (logistic regression) with adjustment for randomization strata. Change in KCCQ-TSS score from baseline to 12 months was modelled as a linear mixed effect model with random effects by participant, an unstructured covariance matrix by treatment group, adjustment for randomization strata and visit by baseline KCCQ-TSS interaction. Missing values were not explicitly imputed so were assumed missing at random. Vague priors [Normal(0, 0.5)] were used for all treatment effect parameters in all secondary efficacy outcomes. Absolute treatment benefit for total HF events (using a negative binomial likelihood) and cardiovascular and all-cause deaths (using a Poisson likelihood) were estimated using counterfactual posterior predictions as described for the primary outcome. We analysed safety outcomes with a binomial likelihood and vague priors, adjusted for randomization strata.

All models were fitted using Hamiltonian Markov Chains (HMC) inference through the R interface to stan, brms.^20,21^ We ran 4 chains for 4000 iterations with a burn-in of 50% for all chains for efficacy outcomes and 2000 iterations for safety outcomes. Model convergence was checked using trace plots, trace-rank plots, R-hat values and autocorrelation plots. Supportive models (negative binomial for recurrent events) were summarized using the marginaleffects package and tidyverse.^22,23^

## Results

There were 6001 patients validly randomized in FINEARTS-HF, 2998 to placebo and 3003 to finerenone. The baseline characteristics have been published previously,^12^ and a condensed version is provided in Supplementary material online, *Table S2*. Patients had a median age of 73 years, and 46% were women. Baseline characteristics were well balanced between the two treatment groups. Most patients (69%) were in NYHA functional class II, and the mean (±SD) left ventricular ejection fraction was 53 ± 8%. The median duration of follow-up was 32 months. For the primary outcome, 624 participants experienced 1083 events in the finerenone arm compared with 1283 events among 719 participants in the placebo arm (see Supplementary material online, *Table S3*). We reproduced the frequentist maximum likelihood estimate of the primary treatment effect, expressed as a rate ratio, as 0.84 with a 95% confidence interval of 0.74 to 0.95 (*p* = 0.007) (see Supplementary material online, *Figure S2*).^9^

### Treatment effect estimates with a vague prior

**Figure 1:**
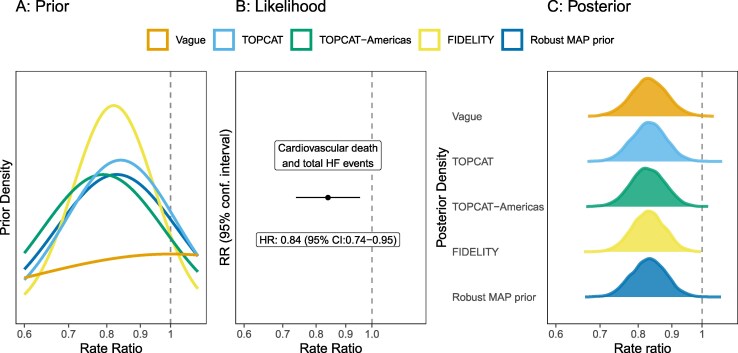
Bayesian estimates of relative treatment benefit with finerenone under different prior distributions. Priors from previous trials applied to FINEARTS-HF (left), estimated treatment benefit from observed data from FINEARTS-HF only (frequentist estimate) (central), Bayesian posterior estimates of the treatment effect of finerenone vs. placebo on the primary composite endpoint of cardiovascular death and total heart failure events under different prior assumptions (right).

Bayesian analysis reflecting no prior belief in the treatment efficacy (vague priors), when combined with the observed trial data, yielded a posterior estimate of the primary rate ratio of 0.83 (95% CrI: 0.74–0.94) (*Figure 1*, *Table 2*) and a posterior probability of treatment efficacy (RR < 1) of 99.85%. The Bayesian models achieved good convergence (see Supplementary material online, *Figures S3–S6*).

**Table 2:** Summary of frequentist and Bayesian posterior estimates of the effect of finerenone vs. placebo for four clinical endpoints

	Estimate (95% CrI)	Posterior probability that treatment is better with finerenone vs. placebo at clinically meaningful thresholds			
**Relative effects**		**<1**	**<0.9**	**<0**.**85**	**<0**.**8**
**CV death and total HF events (rate ratio)**					
Frequentist	0.84 (95% CI: 0.74, 0.95)	—	—	—	—
Vague prior	0.83 (0.74, 0.94)	99.85%	90%	64%	27%
TOPCAT prior	0.83 (0.74, 0.93)	99.88%	92%	65%	26%
TOPCAT-Americas prior	0.83 (0.74, 0.93)	99.91%	92%	67%	29%
FIDELITY prior	0.83 (0.74, 0.92)	>99.99%	93%	67%	27%
Robust MAP prior	0.83 (0.74, 0.93)	99.90%	92%	65%	27%
**Total HF Events (rate ratio)**					
Vague prior	0.82 (0.73, 0.93)	99.96%	93%	71%	33%
**CV Death (hazard ratio)**					
Vague prior	0.93 (0.79, 1.10)	80%	36%	15%	4%
Robust MAP prior	0.92 (0.78, 1.07)	86%	41%	17%	5%
**All Cause Death (hazard ratio)**					
Vague prior	0.94 (0.83, 1.06)	85%	26%	6%	<1%
Robust MAP prior	0.93 (0.83, 1.05)	88%	29%	7%	<1%

Absolute effects (rates and rate reduction per 100 person-years) Vague priors		Posterior probability of fewer events with finerenone	At least 1 fewer event	At least 2 fewer events	At least 3 fewer events
**CV death and total HF events (rate difference)**					
Placebo	23.2 (21, 25.7)				
Finerenone	19.5 (17.5, 21.8)				
Difference	−3.7 (−6.9, −0.7)	99.0%	96%	87%	67%
**Total HF Events (rate difference)**					
Placebo	16.6 (14.9, 18.6)				
Finerenone	13.5 (12, 15.1)				
Difference	−3.2 (−5.5, −0.9)	99.7%	97%	84%	55%
**CV Death (rate difference)**					
Placebo	3.6 (3.2, 4.1)				
Finerenone	3.3 (3, 3.8)				
Difference	−0.3 (−0.8, 0.3)	80%	<1%	<0.1%	<0.1%
**All Cause Death (rate difference)**					
Placebo	7.2 (6.6, 7.9)				
Finerenone	6.8 (6.2, 7.4)				
Difference	−0.5 (−1.3, 0.4)	86%	11%	<0.1%	<0.1%

MAP, meta-analytic prior using data from TOPCAT-Americas and FIDELITY combined. Probabilities shown are the posterior probability that the effect estimate (rate ratio, hazard ratio or rate difference) is less than the specified threshold.

Bayesian posterior distributions can be evaluated at alternative critical values to the null. We estimated the probability that finerenone reduced the rate of cardiovascular death and total HF events by at least 10% (equivalent to RR < 0.9) as 90%, by 15% (RR < 0.85) as 64% and by more than 20% (RR < 0.80) as 27% (*Table 2*).

### Treatment effect estimates with informative priors

Using informative priors from a single historical trial assigned a high prior probability of finding a beneficial treatment effect (*Table 1*), and correspondingly, the posterior probability of greater treatment effects was slightly increased with these priors. The probability of a relative benefit greater than 10% [Pr(RR < 0.9)] when informed by TOPCAT (92%), TOPCAT Americas (92%) or FIDELITY (93%) was larger than using a vague prior (90%) (*Table 2*). Using historical results without downweighing as priors increased both the prior and posterior probabilities of a treatment benefit with finerenone (see Supplementary material online, *Table S4*).

Pooling the results from FIDELITY and TOPCAT Americas in a robust MAP prior incorporated all this prior information and variation between them. This also slightly increased the posterior probability of a larger treatment benefit compared to the estimate using vague priors [Pr(RR < 0.9) = 92%] (*Table 2*). These findings were consistent when including TOPCAT Americas or all TOPCAT in the MAP prior derivation (see Supplementary material online, *Figure S7*).

### Absolute and relative benefits of finerenone on heart failure hospitalizations and mortality

To maximize the benefits of Bayesian models’ flexibility and their ability to summarize quantities in clinically intuitive terms, we analysed the effect of finerenone using negative binomial and Poisson models. For the primary outcome, using vague priors and a negative binomial model likelihood, we estimated a similar rate ratio as the LWYY model (0.84, 95% CrI: 0.73–0.97). This same model can also be used to estimate the treatment effect on an absolute scale. Using this method, we estimated an event rate in the placebo arm of 23.2 events per 100 person-years (95% CrI: 21.0, 25.7) and 19.5 events with finerenone (95% CrI: 17.5, 21.8), for a rate difference of 3.7 fewer events per 100 patient-years (95% CrI: 0.7, 6.9) (see Supplementary material online, *Figure S8*). Similarly, for total HF events excluding mortality, we estimated a high probability of a benefit on the relative scale (RR, 0.82; 95% CrI: 0.73, 0.93) and on an absolute scale (rate difference, 3.2 fewer events; 95% CrI: 0.9, 5.5) (*Table 2*, Supplementary material online, *Figure S9*).

**Figure 2:**
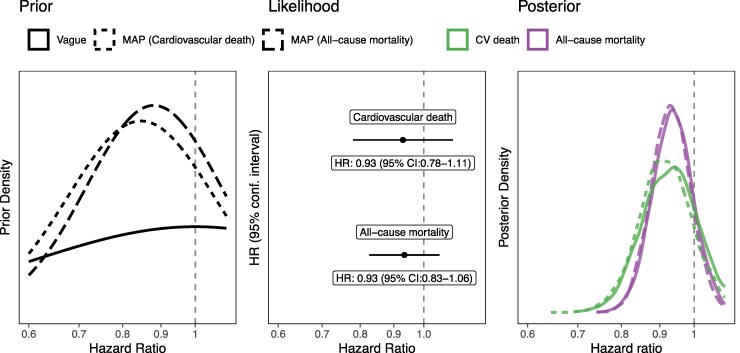
Bayesian estimates of relative treatment benefit with finerenone under different prior distributions on mortality. Priors from previous trials applied to FINEARTS-HF (left), estimated treatment benefit from observed data from FINEARTS-HF only (frequentist estimate) (central), Bayesian posterior estimates of the treatment effect of finerenone vs. placebo on the mortality endpoints of cardiovascular death and all-cause mortality (right).

The frequentist analysis of mortality outcomes in FINEARTS-HF was unable to reject the null hypothesis of no difference in cardiovascular (HR 0.93; 95% CI: 0.78, 1.11) or all-cause mortality (HR 0.93; 95% CI: 0.83, 1.06) (see Supplementary material online, *Table S3*). Using a vague prior, we estimated an 80% posterior probability that the finerenone vs. placebo hazard ratio for CV death was less than one (i.e. favours finerenone) and an 85% probability of treatment benefit on all-cause death (*Figure 2*, *Table 2*). We also incorporated priors derived from FIDELITY and TOPCAT-Americas to define a meta-analytic prior for each outcome (see Supplementary material online, *Figure S10*). These prior distributions remained broad but assigned more probability to a treatment benefit than harm on both CV and all-cause mortality (see Supplementary material online, *Figure S10*). The posterior probability of any treatment benefit [Pr(HR < 1.0)] with finerenone therefore, increased to 86% for CV death, and 88% for all-cause mortality (*Table 2*).

**Figure 3:**
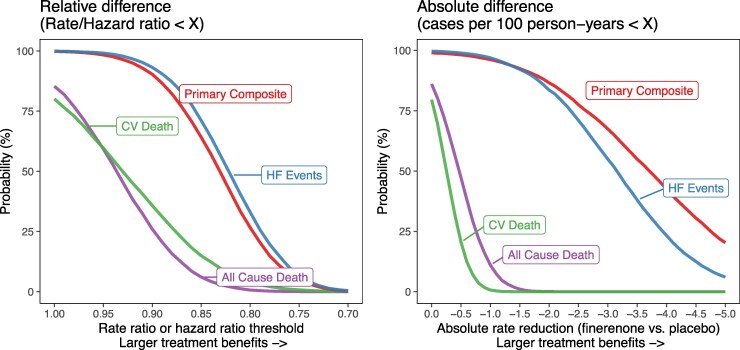
Probability threshold curves for relative and absolute treatment benefit with finerenone. Estimates of the posterior probability of a relative benefit (rate ratio or hazard ratio) and absolute benefit (rate difference per 100 person-years) with finerenone on four major cardiovascular clinical endpoints.

Our Bayesian analysis means that we can calculate the posterior probability of varying magnitudes of treatment benefits on both relative and absolute scales. *Figure 3* shows these posterior probabilities, using vague priors, for four clinical endpoints and shows a higher probability of benefit for endpoints including HF events. There was a slightly higher probability of a relative benefit for total HF events alone, but a higher probability of greater benefits on an absolute scale for the composite of cardiovascular death and total HF events. The relatively high probability of benefit for mortality endpoints declines rapidly for larger treatment benefits, especially on an absolute scale.

Using a vague prior for the treatment effect, we derived very similar estimates using Bayesian analyses to those found in the frequentist analyses of all secondary outcomes (see Supplementary material online, *Figure S11*, Supplementary material online, *Table S3*).

### Effect of finerenone on safety outcomes

Safety outcomes were also analysed in a Bayesian framework using logistic regression and vague priors, which enabled these outcomes to be summarized on both a relative and absolute (see Supplementary material online, *Table S5*). There was no difference in reported serious adverse events (OR 1, 95% CrI: 0.9–1.1). Finerenone increased the probability of a potassium level above 5.5 mmol/L from 7.1% in the placebo arm to 14.6% in the finerenone arm, an adjusted risk difference of 7.1% (95% CrI: 5.6–8.7), but decreased the probability of a potassium level below 3.5 mmol/L by 4.9% (95% CrI: 3.6–6.2). Finerenone increased the probability of a systolic blood pressure measurement of less than 100 mmHg [adjusted risk difference of 5.6% (95% CrI: 4.0–7.1)] and of a creatinine level of 2.5 mg/dL or more [adjusted risk difference of 1.8% (95% CrI: 0.7–2.8)].

## Discussion

In this Bayesian analysis of the FINEARTS-HF trial, we found that the probability that finerenone reduced the rate of cardiovascular death and total HF events was 99.85% and that the probabilities of at least 10%, 15%, and 20% relative risk reductions were 90%, 64%, and 27%, respectively, assuming no meaningful prior information. Incorporation of prior data from the previous finerenone and spironolactone trials supported this conclusion [Pr(RR < 1) = 99.90%, Pr(RR < 0.9) = 92%, Pr(RR < 0.85) = 65%, Pr(RR < 0.8)= 27%], respectively, using a robust meta-analytic prior. For context, the updated level of evidence grading system for ESC clinical practice guidelines recommends level A for ‘substantial evidence against the play of chance’, defined as *P* < 0.005, which approximately corresponds to a posterior probability of superiority of 99.75% if minimally informative priors are used.^24^ We also estimated an 80% probability that finerenone reduced the risk of cardiovascular death (i.e. that the hazard ratio was <1.0) and an 85% probability that it reduced all-cause death using vague priors.

Bayesian analyses can supplement frequentist approaches, which are the most commonly used analyses of randomized clinical trials.^2,3,25,26^ We have demonstrated several of the advantages of Bayesian approaches using the results of the FINEARTS-HF trial. Without the binary reliance on null hypothesis significance testing of frequentist methods,^27-30^ a Bayesian approach enabled us to make probabilistic statements about treatment benefit at multiple clinically relevant thresholds. In addition to the frequentist analysis of FINEARTS-HF, this analysis has provided additional data on the probability of various relative treatment benefits, quantified the probability of a benefit on all cause death, estimated absolute benefits with finerenone vs. placebo, and importantly, incorporated previous knowledge regarding MRAs in patients with HFmrEF/HFpEF into the results of the FINEARTS-HF trial.

We were able to quantify and incorporate prior information on both the effect of finerenone on cardiovascular outcomes in the FIDELIO-DKD and FIGARO-DKD trials^10,11^ and the effect of a steroidal MRA in patients with HFmrEF/HFpEF enrolled in the TOPCAT trial.^5,6^ We used the most agnostic and vague prior for our primary analysis and calculated a 90% probability that the treatment effect was at least 10%. We have demonstrated that this conclusion of treatment benefit with finerenone is consistent or enhanced across a range of plausible prior beliefs. If we used the most optimistic prior based on the treatment effect from the patients enrolled in the FIDELITY pooled trials, the posterior probability of a treatment effect of at least 10% increased to 93%. The meta-analytic prior method provides a robust framework with which to combine prior information from multiple studies without exerting undue influence. With this approach, we estimated an improved posterior probability of 92% for a 10% reduction in the relative rate of cardiovascular death and total HF events.

Consistent with previous trials of patients with HFmrEF/HFpEF, frequentist analysis of FINEARTS-HF did not show evidence of a reduction in cardiovascular or all-cause mortality.^31-36^ A meta-analysis of TOPCAT and FINEARTS-HF was also unable to show a reduction in cardiovascular or all-cause mortality.^15^ We were able to expand on these previous trials in the Bayesian framework by quantifying the probability that finerenone did reduce the hazard of cardiovascular death (80%) and all-cause mortality (85%) using vague priors. These probabilities were higher if we used informative priors from FIDELITY and TOPCAT-Americas through a MAP prior (86% and 88%, respectively). Even with this stronger prior belief, the probability of a large relative treatment benefit on mortality was low, for example the probability of an HR < 0.9 for CV mortality was 41% and on an absolute scale the probability of 1 fewer CV death per 100 person-years was <1%.

This enables clinicians and regulators to judge the strength of evidence for a treatment benefit in context, instead of rejecting conclusions based on failure to meet a certain statistical significance threshold. The relatively low mortality rates and higher incidence of non-cardiovascular causes of death in HFmrEF/HFpEF compared to HFrEF mean that a trial would need to be unfeasibly large to realistically demonstrate a mortality treatment benefit under a frequentist statistical analysis plan.^37^ Bayesian methods therefore, provide a valuable pathway to better understand treatment efficacy on mortality in this patient population.

A crucial unanswered question for future research is whether incorporating robust and informative prior information can reduce the size of new trials in HF when using Bayesian analyses. Combining Bayesian data analysis with adaptive trial design may further improve efficiency.^38-40^ However, the desire to reduce trial sample size and cost must be balanced against regulatory requirements that preserve frequentist operating characteristics and overcome scepticism about subjective prior definition.^41^ Trials must also be large enough to provide a robust evaluation of safety endpoints.

Our study has some limitations. The sources of prior information were from slightly different patient populations and from steroidal and non-steroidal mineralocorticoid receptor antagonists, which may affect the assumption of exchangeability between these trials. For this reason, we excluded this information in our primary analysis and used vague priors, but we also found consistent results across a wide range of prior specifications in our secondary analyses which suggests that deriving priors from slightly different patient populations or treatments did not alter our conclusions. Secondly, we were unable to incorporate data on eplerenone into our priors as the only trial of eplerenone was conducted in patients with HFrEF.^42^ In a prior meta-analysis, the treatment effect of MRAs there was significant heterogeneity in the treatment effect of MRAs in the trials of patients with HFrEF compared to HFmrEF/HFpEF^15^ precluding inclusion in this analysis. Thirdly, we have included alternative statistical models to the primary FINEARTS-HF frequentist analysis with negative binomial and Poisson models.^9^ This allowed us to quantify absolute treatment effects, which is challenging with the semi-parametric survival models. While the specific details of prior specification and analysis strategy were not pre-registered our analysis was pre-specified.

In this Bayesian analysis of FINEARTS-HF, finerenone reduced the risk of cardiovascular death and total HF events. The analysis showed that there is a > 80% probability that finerenone caused a small reduction in CV death and all-cause mortality.

## Supplementary Material

pvag010_Supplementary_Data

## Data Availability

Data will be made available to qualified scientific and medical researchers through vivli.org. All requests will be reviewed by an independent scientific review panel and data provided according to the conditions laid out on https://vivli.org/ourmember/bayer/.
