# Supplementary material for: A Bayesian analysis of finerenone in heart failure with mildly reduced and preserved ejection fraction: a pre-specified analysis of FINEARTS-HF
Source: Eur Heart J Cardiovasc Pharmacother. 2026 Feb 16;12(3):176–85. doi: 10.1093/ehjcvp/pvag010 (PMC13185745; doi:10.1093/ehjcvp/pvag010)
Supplement: pvag010_Supplementary_Data [file pvag010_supplementary_data.pdf]

# A Bayesian analysis of finerenone in heart failure with mildly reduced and preserved ejection fraction: a pre-specified analysis of FINEARTS-HF

Alasdair D Henderson[1], Kieran F Docherty[1], Atefeh Talebi[1], Toru Kondo[1,2], Mark C Petrie[1], Brian L Claggett[3], Akshay S Desai[3], Muthiah Vaduganathan[3], John J Atherton[4], Johan Bauersachs[5], Morten Schou[6], Subodh Verma[7], Carolyn SP Lam[8], Bertram Pitt[9], Michele Senni[10], Sanjiv J Shah[11], Adriaan A Voors[12], Faiez Zannad[13], Meike Brinker[14], Flaviana Amarante[15], Katja Rohwedder[16], James Lay-Flurrie[17], Scott D Solomon[3], John JV McMurray[1], Pardeep S Jhund[1]

# Supplementary Materials

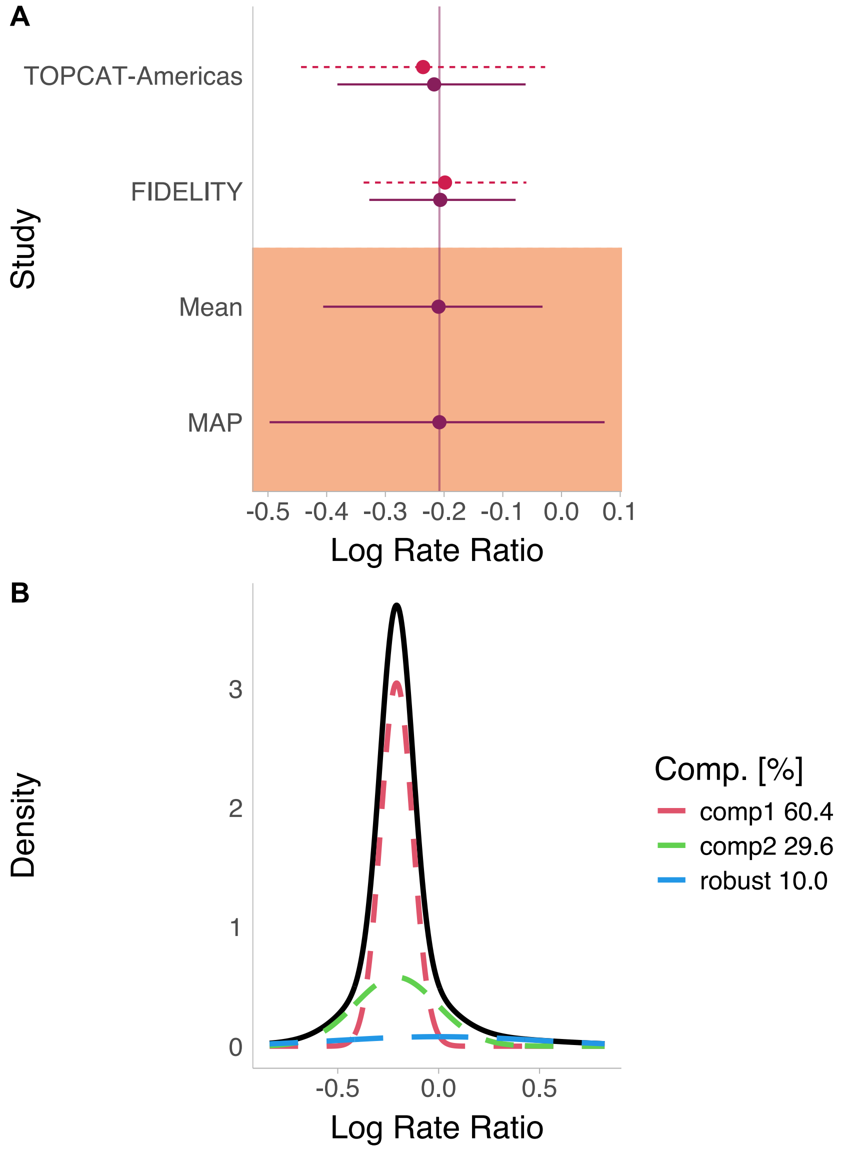


*Supplementary Figure 1: Summary of meta-analytic predictive prior. A) Results from the Bayesian meta-analysis of a previous placebo-controlled randomised trials of a steroidal MRA in HFmrEF/HFpEF (TOPCAT-Americas) and of finerenone (FIDELITY). B) summary of mixed normal distribution used as the meta-analytic prior. The final prior (black line) is a mixture of five components where the purple line ("robust") represents a 10% contribution from the same vague prior as used in the primary analysis ~N(0, 0.5)*

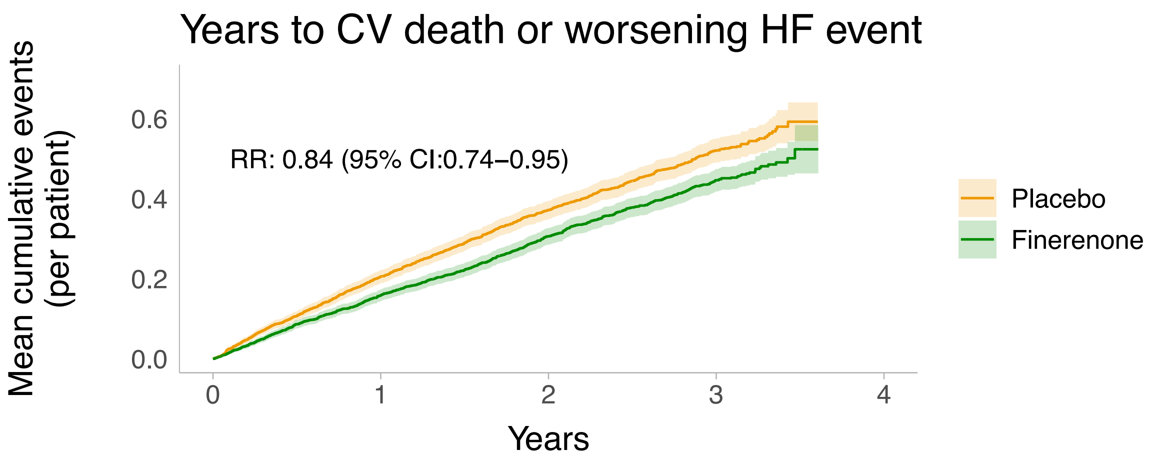


| | | | | | |
|---|---|---|---|---|---|
| **Placebo** | | | | | |
| At Risk | 2998 | 2802 | 2179 | 848 | 0 |
| Events | 0 | 596 | 1020 | 1261 | 1283 |
| **Finerenone** | | | | | |
| At Risk | 3003 | 2804 | 2184 | 841 | 0 |
| Events | 0 | 461 | 833 | 1063 | 1083 |

*Supplementary Figure 2: Frequentist estimate of the primary treatment effect of finerenone reducing the rate of CV death or first and recurrent worsening HF events. Survival curves show the mean cumulative events (Nelson-Aalen cumulative hazard estimates)*

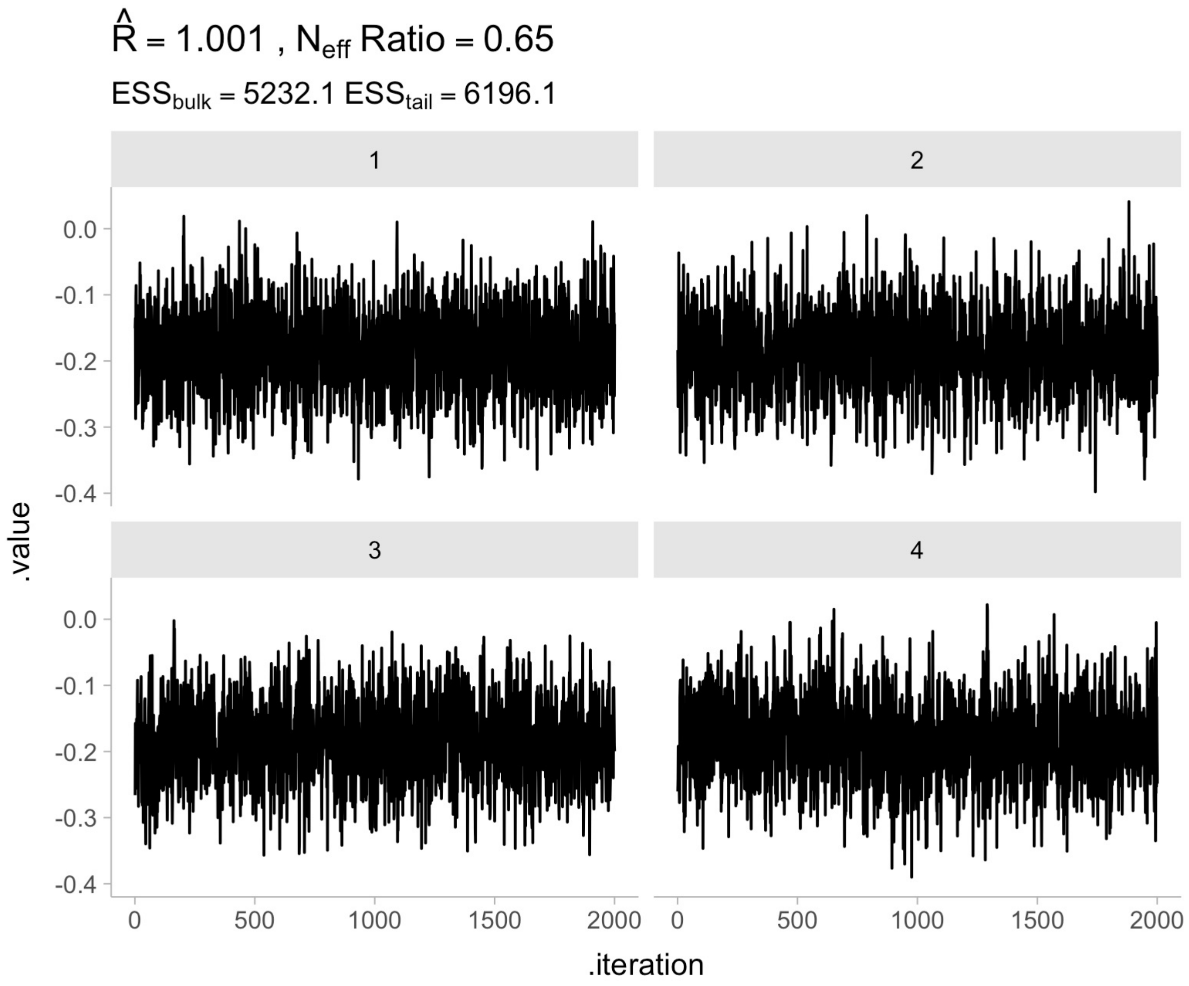


*Supplementary Figure 3: Trace plots for the primary analysis of first (and recurrent) worsening HF events and CV death, using a vague prior. Trace plots are displayed for the samples from four chains withn 4,000 iterations in total and a burn-in of 2,000 iterations*

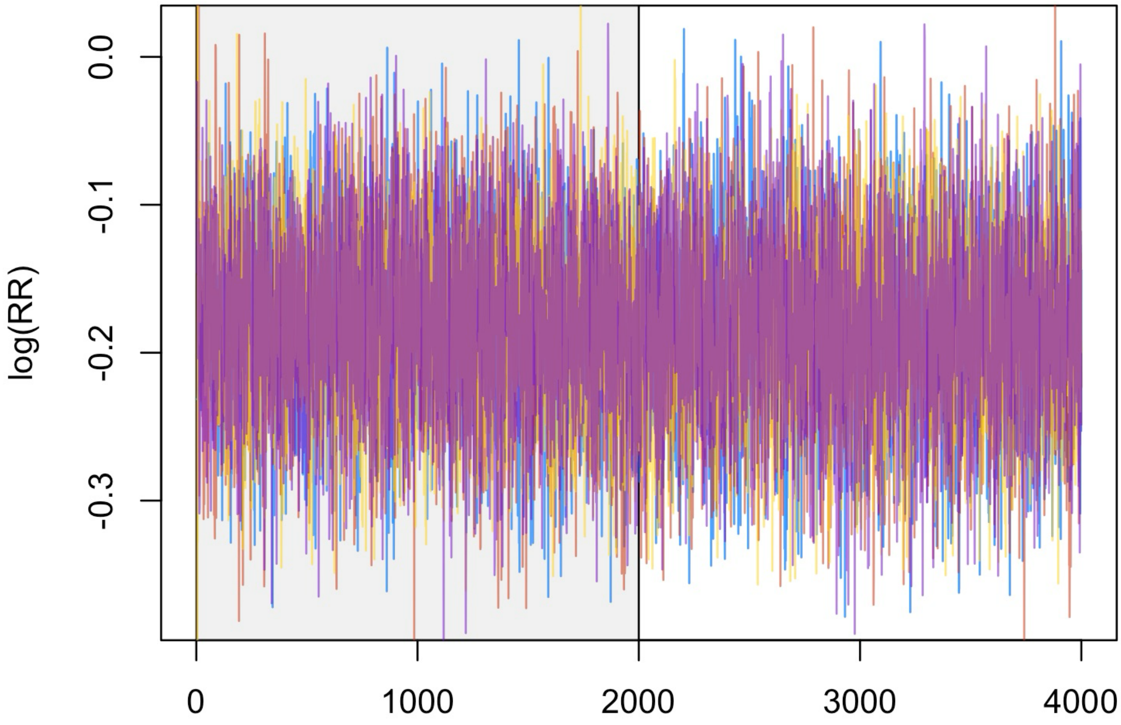


*Supplementary Figure 4: Overlayed trace plots from the four chains used in the primary model (with vague prior). The shaded region from 0 to 2,000 displays the burn-in period and discarded samples from the posterior chains*

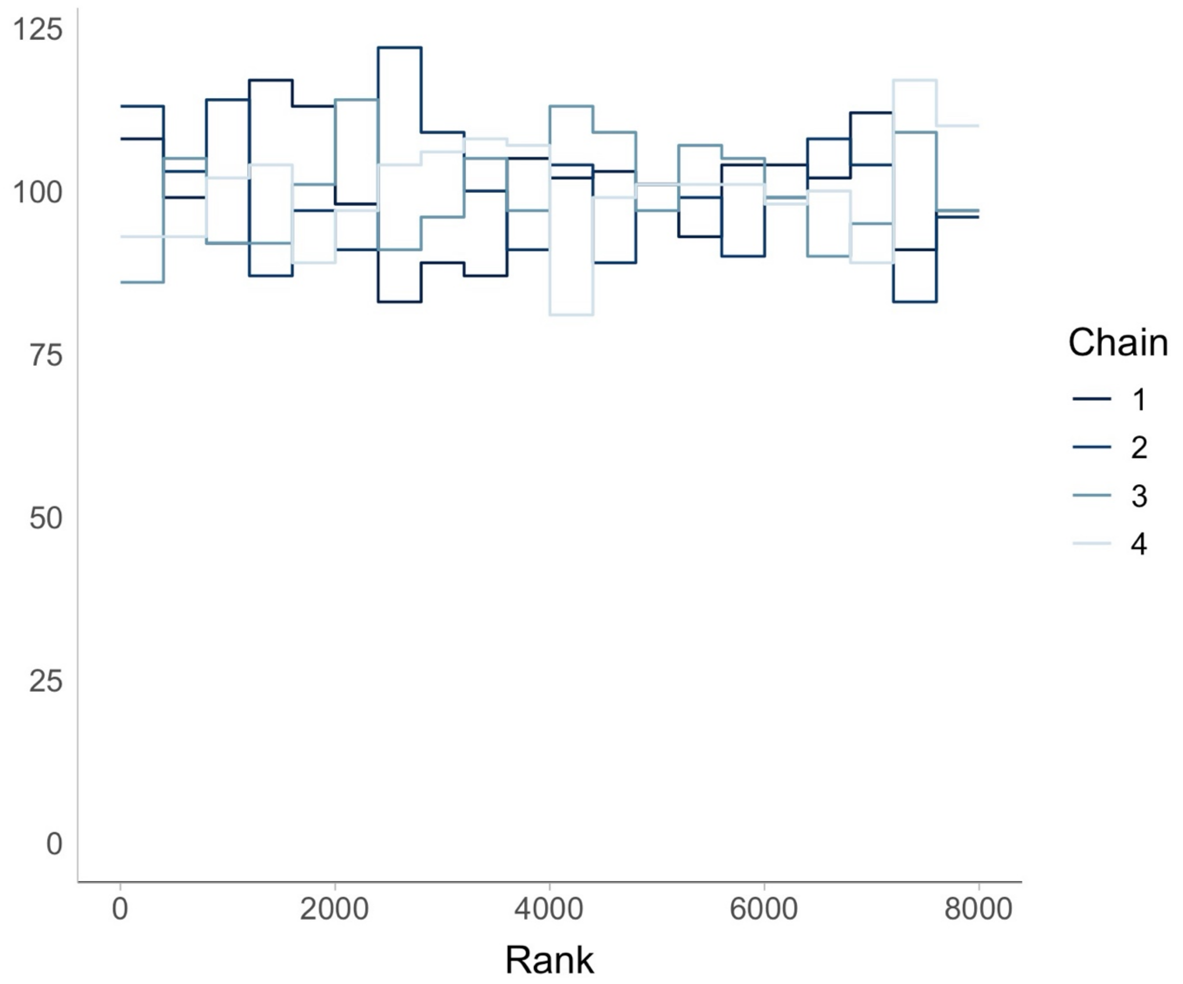


*Supplementary Figure 5: Trace-rank plots of the four chains from the primary model (with vague priors) for the primary outcome (first and recurrent worsening HF events and CV death)*

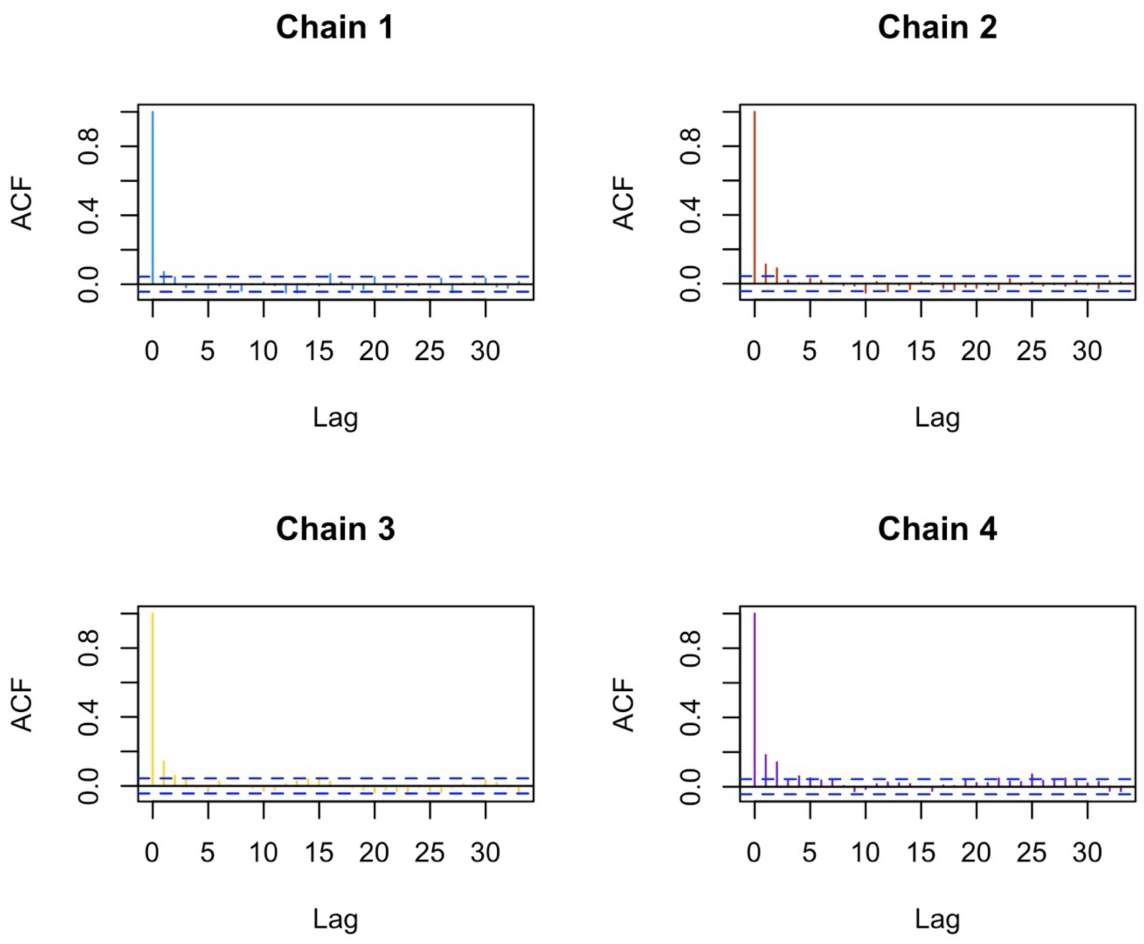


*Supplementary Figure 6: Estimates of the auto-correlation function of the posterior chains for the primary outcome model*

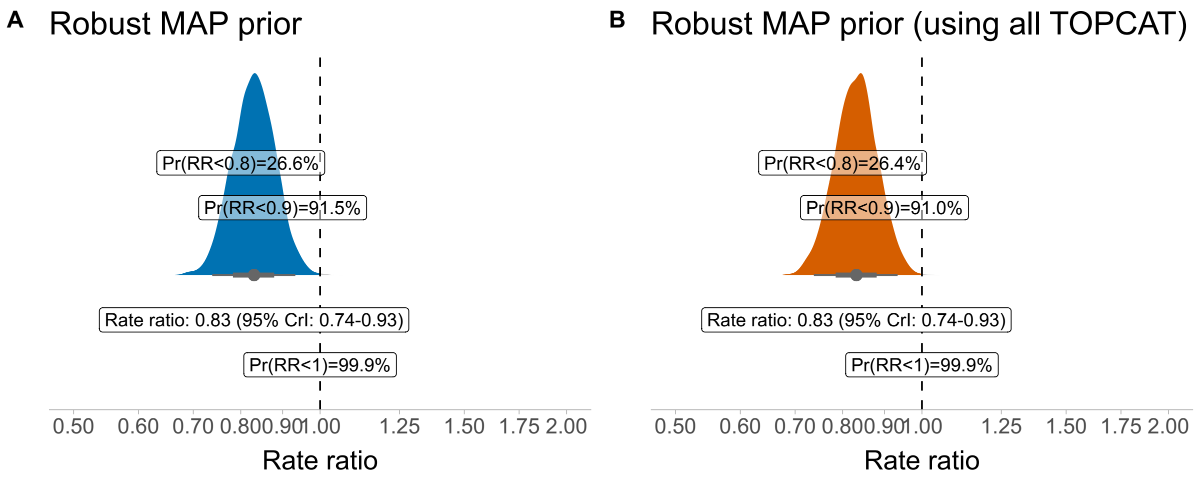


*Supplementary Figure 7: Comparison of posterior distributions incorporating TOPCAT data. A) TOPCAT Americas data were combined with FIDELITY to derive the MAP prior. B) all data from TOPCAT was used. The overall change in posterior probability of any treatment benefit was 0.05%*

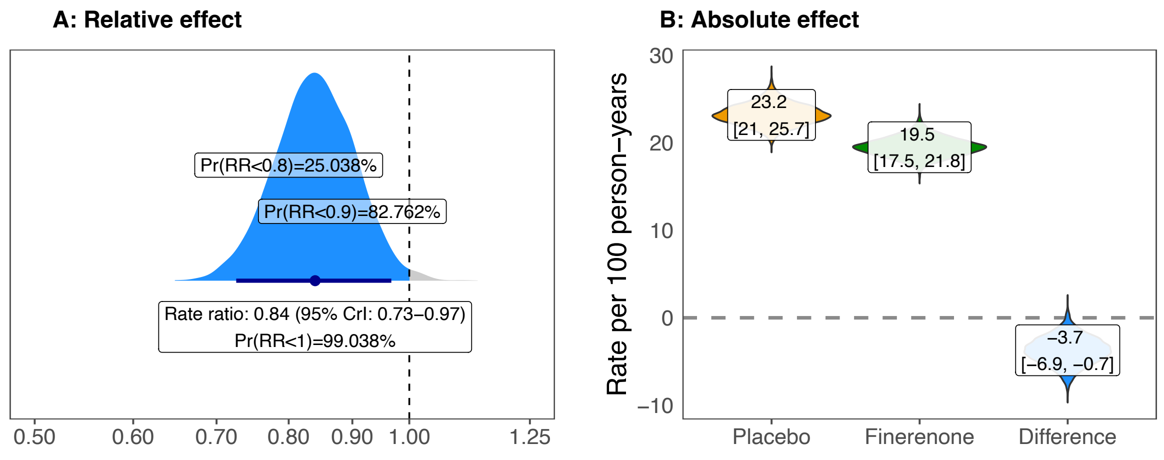


*Supplementary Figure 8: Summary of Bayesian negative binomial model of treatment benefit. Estimates of the relative (A) and absolute (B) efficacy of finerenone on reducing the rate of total worsening heart failure events and cardiovascular death versus placebo*

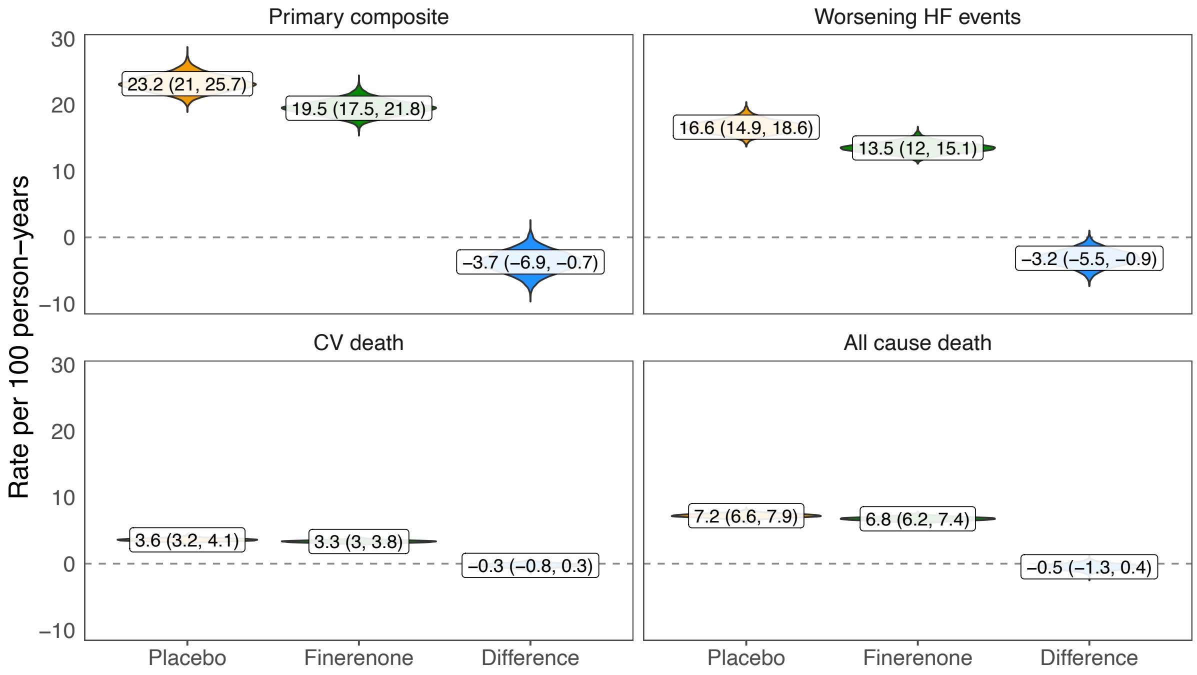


*Supplementary Figure 9: Rates and rate differences per 100 person-years for mortality and heart failure hospitalization endpoints. Rates are estimated from Bayesian models using negative binomial likelihood (primary composite and total worsening HF events) or Poisson likelihood (CV death and all cause death), by estimating counterfactual predicted events for 1 year of follow up for each treatment group and taking their difference.*

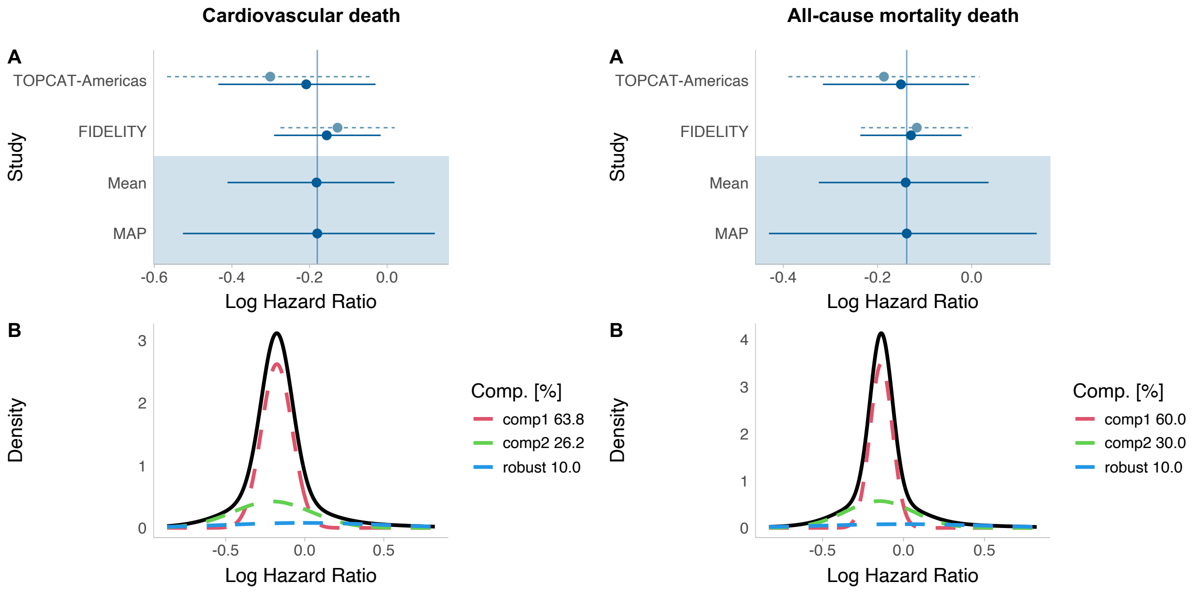


*Supplementary Figure 10: Meta-analytic priors for the two mortality outcomes, derived from estimates of the effect of spironolactone (from TOPCAT-Americas) and finerenone (from FIDELITY) on the hazard of CV death (left column) or all-cause mortality (right column).*

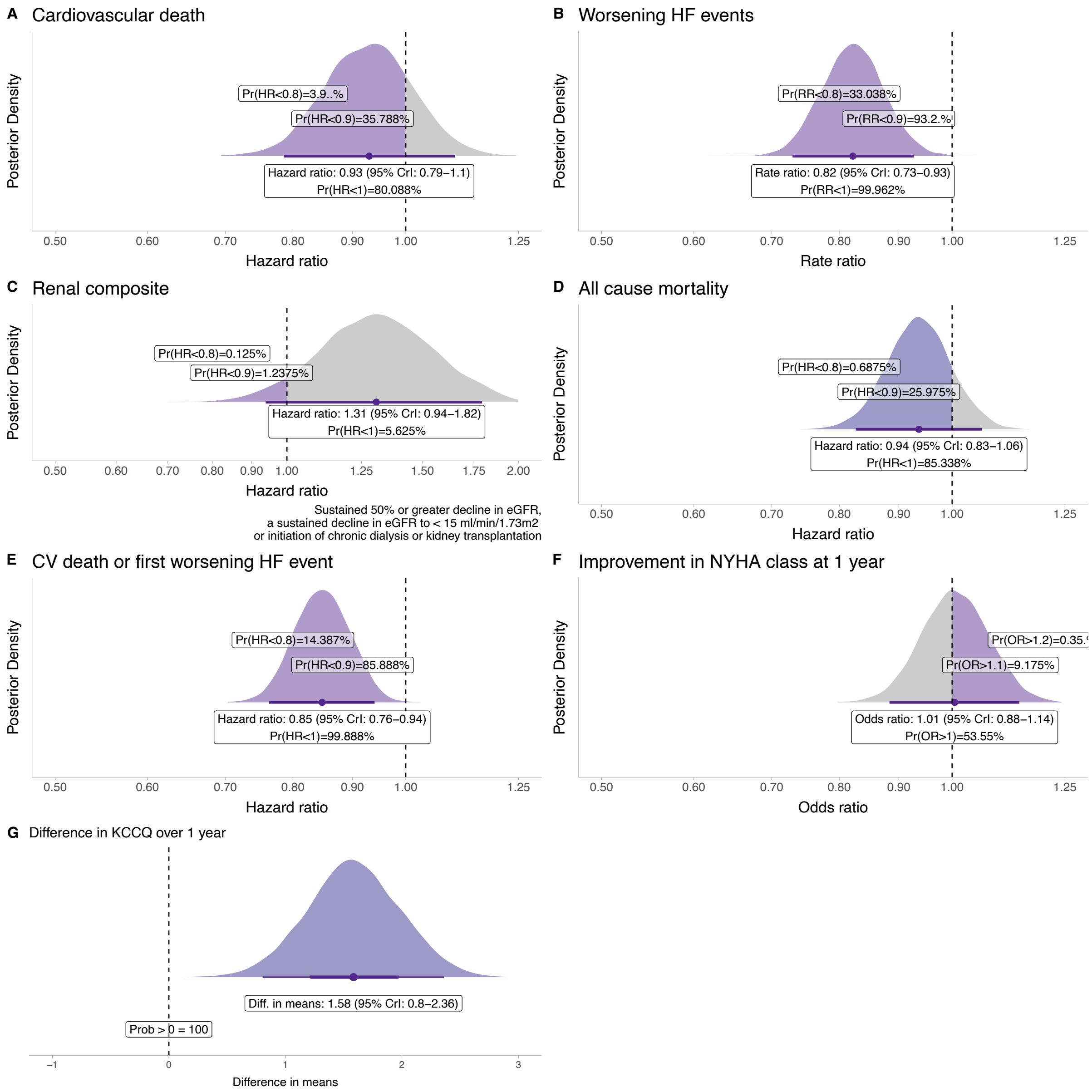


*Supplementary Figure 11: Posterior distributions from Bayesian analyses of all secondary outcomes included in the FINEARTS-HF trial. All models used default vague priors from the {brms} package in R, except treatment effect (log hazard or rate ratio) which used a Normal(0, 0.5) prior. Time to event outcomes (panels A, C, D, and E) used stratified Cox models. Recurrent worsening HF events (panel B) used a semi-parametric LWYY survival model as in the primary analysis. Change in NYHA used a logistic regression and mean change in KCCQ used a mixed effects model. All models were either stratified or adjusted for randomisation strata (region and LVEF above/below 60%)*

| Guideline | Detail of how it was addressed in the article |
|---|---|
| **BayesWatch: Bayesian methods in health technology assessment: a review** | |
| *Introduction* | |
| 1. The technology: The intervention to be evaluated must be clearly described with regard to the population of interest and other relevant details. | Intervention is finerenone, a non-steroidal mineralicorticoid antagonist. Population is adults with symptomatic HFmrEF/HFpEF. Key inclusion and exclusion criteria are described |
| 2. Objectives of study: Distinguish between desired inferences on quantities of interest (parameters to be estimated) and decisions or recommendations for action. The former requires a prior distribution, the latter a loss function/utility. | Estimate the effect relative and absolute effect of finerenone versus placebo on the rate of worsening HF events and cardiovascular death |
| *Methods* | |
| 1. Design of study: Ensure similarity of studies to justify assumptions of exchangeability when synthesizing evidence. | Prospective, randomized, double-blind, placebo-controlled clinical trial |
| 2. Statistical model: Describe the probabilistic relationship between the parameter(s) of interest and the observed data, either mathematically or in a way that allows its mathematical form to be unambiguously obtained. | Primary outcome: Semi-parameteric proportional hazards model using LWYY, stratified with specific baseline hazards estimated per strata. Supportive analysis: Negative binomial model. Secondary efficacy and safety outcomes: LWYY, Cox proportional hazards model, logistic regression and mixed-effect multilvel model. |
| 3. Prospective analysis?: Clarify whether the prior and any loss function were constructed before data collection and whether analysis was carried out during the study. | Partially. Design and sources of information for prior distributions were pre-registered before the close of the trial. The analysis was conducted retrospectively |
| 4. Loss function: State if an explicit method of deducing scientific consequences was decided prior to the study, including any range of equivalence or loss function. Describe the elicitation process if used. | None |
| 5. Prior distribution: Provide explicit priors for the parameters of interest, indicating whether they are informative or non-informative. Describe the elicitation process and any empirical evidence underlying the prior assessment. | Vague and informative priors are used and described in full. The main results were derived using a Normal(0, 0.5) prior on the log(rate ratio) scale. Further informative priors were derived from previously published rate ratios, hazard ratios, and meta-analytic predictive priors derived from these published results |
| 6. Computations: Describe computations in detail, including software used, MCMC methods, starting values, number and length of runs, and convergence diagnostics. | HMC using brms in R. We ran 4 chains for 4,000 iterations with a burn-in of 50% for all chains. We used 8,000 iterations for subgroup models to ensure convergence |
| *Results* | |
| 1. Evidence from study: Provide as much information about the observed data as possible, including sample sizes and measurements taken, while maintaining brevity and data confidentiality. | Primary analysis: 624 participants experienced 1083 events in the finerenone arm compared with 1283 events among 719 participants in the placebo arm. The frequentist likelihood estimate expressed as a rate ratio was 0.84 with a 95% confidence interval of 0.74 to 0.95 |
| *Interpretation* | |
| 1. Reporting: Summarize posterior distributions, including credible intervals and graphical presentations. Distinguish between current summaries for action and contributions of information for future action. | Posterior distributuions are presented and 95% Credible intervals are provided throughout |
| 2. Sensitivity analysis: Present results of alternative priors and/or expressions of the consequences of decisions. | Different prior specifications were used for the primary efficacy outcome and for secondary mortality outcomes. Vague prirors were used for |

| | |
|---|---|
| | main resutls, but informative priors derived from previously published trials were also used. Sensitivity anlaysis was also provided through the use of a different statistical model (Negative binomial) |
| **Key Standards for Conducting, Reporting, and Interpreting Results of Bayesian Analysis** | |
| Specify choice of prior distribution (normal, uniform, binomial, gamma) and the hyperparameter values (eg, mean, variance for a normal distribution), as well as the rationale and justification for this choice. | Vague, normally distributed priors are used for the amin treatment effects on the log rate ratio scale. Informative priors are used as supportive analyses to examine the influence of incorporating results from previous trials with the same or similar medication in the same or similar populations |
| If an informative prior distribution is used, reviewers are likely to look with extra scrutiny, so authors should give a strong rationale for the choice of an informative prior and justification for the specific choices made. | Details of the source of prior information are provided in the methods. We only used results from a trial of a similar medication in the same targetted HFmrEF/HFpEF population, or trials of the same medication in similar targetted populations with cardiometabolic disease. Only results from randomised, placebo-controlled trials were used as informative priors |
| Specify the structure of higher levels of the model, in the case of hierarchical models. | Random effects by participant were used for mixed effects models of KCCQ-TSS as a secondary endpoint |
| If the analysis will potentially be used to support a regulatory decision in support of treatment efficacy, it may be appropriate to define a success criterion (eg, posterior probability of effect >0.95 or another threshold) and to demonstrate by simulation that the approach retains frequentist type I error of no greater than 2.5% or 5%. | Not applicable |
| Methods for generating posterior summaries, including analytical technique or simulation such as Markov Chain Monte Carlo method and software used for analysis. | Hamiltonian markov chains were used to derive posterior summaries. Full details on specification and software are provided |
| Present numeric or graphical summaries of the prior distribution and the posterior distribution. | Figures 1 and 3 show prior distributions. Figures 1-3 show posterior distributions for the various models |
| Report posterior mean or median and appropriate credible interval for parameters or effects of interest. | All results report median, 95% credible intervals and are shown graphically with interval ranges |
| Report posterior probabilities of effect sizes of interest, for example, P(hazard ratio >1) for effect >0 or P(hazard ratio >1.2) if that is considered a clinically relevant effect of interest. | Posterior probabilities are reported and highlighted throughout the results section |
| Discuss robustness to different choices of prior distributions. | Figures 1 and 3 show the effect of different prior distributions and these are discussed at length |
| **WAMBS-v2: Bayesian statistics and modelling** | |
| Ensure the prior distributions and the model (or likelihood) are well understood and described in detail in the text, including the hyperparameter settings and all details surrounding the model. In addition, prior-predictive checking can help identify any prior-data conflict. | Full model specification in the Methods. Code available in the appendix. |
| Assess each parameter for convergence. Use multiple convergence diagnostics if possible. This may involve examining trace-plots or ensuring diagnostics (e.g., $\hat{R}$ or effective sample size) are being met for each parameter. For example, $\hat{R}$ values smaller than 1.05 are typically recommended. Likewise, effective sample sizes of 10,000 or more are recommended as a general rule of thumb. | Convergence of primary results presented in the appendix (suppl. Figure 3) showing good convergence of all chains. The same diagnostics were run for all models but are not presented here for issues of space. |

| | |
|---|---|
| Sometimes convergence diagnostics can fail at detecting non-convergence within the chain. Subsequent measures, such as the split-$\widehat{R}$ can be used to identify such situations. The split-$\widehat{R}$ can detect trends that are missed if the chains have similar marginal distributions (the $\widehat{R}$ may miss these trends). | Not performed |
| Ensure that there were sufficient chain iterations to construct a meaningful posterior distribution. The posterior distribution should consist of enough samples to visually examine the shape, scale, and central tendency of the distribution. Without enough samples, there is an incomplete picture of the full distribution. | Confirmed and full posterior distributions are graphically presented throughout the manuscript |
| Check all parameters for strong degrees of autocorrelation (e.g., through examining the effective sample size for parameters), which may be a sign of model or prior misspecification. | Autocorrelation plot provided for primary results model showing no major concerns (Supplementary Figure 6) |
| Visually examine the marginal posteriors distribution for each model parameter to ensure that they make substantive sense. Posterior predictive distributions can be used to aid in examining the posteriors. | Confirmed and full posterior distributions are graphically presented throughout the manuscript |
| Fully examine multivariate priors through a sensitivity analysis. These priors can be particularly influential on the posterior, even with slight modifications to the hyperparameters. | Multivariate priors are not used in these analyses. Multiple priors were used and compared to assess sensitivity of prior specification to inferences throughout the article. |
| To fully understand the impact of subjective priors, compare the posterior results to an analysis using diffuse (or objective) priors. This comparison can facilitate a deeper understanding of the impact the subjective priors (i.e., the theory being implemented) are having on findings. Next, conduct a full sensitivity analysis of all priors to gain a clearer understanding of the robustness of the results to different prior settings. | Performed and presented throughout the manuscript |
| Given the subjectivity of the model, it is also important to conduct a sensitivity analysis of the model (or likelihood) to help uncover how robust results are to deviations in the model. | Model (likelihood) was specified in the primary statistical analysis plan and this re-analysis used the same model as the primary frequentist analyses to facilitate comparison between the two underpinning statistical methodologies |
| Report findings by including Bayesian interpretations. Take advantage of explaining and capturing the entire posterior rather than simply a point estimate. For example, it may be helpful to examine the density at different quantiles to fully capture and understand the posterior distribution. | Performed and presented throughout the manuscript |

*Supplementary Table 1: Bayesian reporting checklists*

| Characteristic | Overall, N = 6,001[1] | Placebo, N = 2,998[1] | Finerenone, N = 3,003[1] |
|---|---|---|---|
| Sex | | | |
| Male | 3,269 (54%) | 1,621 (54%) | 1,648 (55%) |
| Female | 2,732 (46%) | 1,377 (46%) | 1,355 (45%) |
| Age | 73 (66, 79) | 73 (66, 79) | 73 (66, 79) |
| Pooled Race Group | | | |
| White | 4,735 (79%) | 2,369 (79%) | 2,366 (79%) |
| Black | 88 (1.5%) | 39 (1.3%) | 49 (1.6%) |
| Asian | 996 (17%) | 499 (17%) | 497 (17%) |
| Other | 182 (3.0%) | 91 (3.0%) | 91 (3.0%) |
| Region | | | |
| Western Europe, Oceania and Others | 1,256 (21%) | 632 (21%) | 624 (21%) |
| Eastern Europe | 2,650 (44%) | 1,321 (44%) | 1,329 (44%) |
| Asia | 983 (16%) | 490 (16%) | 493 (16%) |
| North America | 471 (7.8%) | 236 (7.9%) | 235 (7.8%) |
| Latin America | 641 (11%) | 319 (11%) | 322 (11%) |
| History of heart failure hospitalization | | | |
| No previous HFH | 2,382 (40%) | 1,176 (39%) | 1,206 (40%) |
| Previous/ongoing HFH at randomisation | 3,619 (60%) | 1,822 (61%) | 1,797 (60%) |
| Baseline eGFR (mL/min/1.73m2) | 61 (47, 77) | 61 (47, 78) | 61 (47, 76) |
| Baseline eGFR (mL/min/1.73m2) | | | |
| < 60 mL/min/1.73m2 | 2,888 (48%) | 1,437 (48%) | 1,451 (48%) |
| >= 60 mL/min/1.73m2 | 3,113 (52%) | 1,561 (52%) | 1,552 (52%) |
| Baseline LVEF (%) (Mean ± std. dev.) | 52.6 ± 7.8 | 52.5 ± 7.8 | 52.6 ± 7.8 |
| Baseline LVEF categories | | | |
| < 50% | 2,172 (36%) | 1,079 (36%) | 1,093 (36%) |
| >= 50% - < 60% | 2,674 (45%) | 1,345 (45%) | 1,329 (44%) |
| >= 60% | 1,147 (19%) | 572 (19%) | 575 (19%) |
| Unknown | 8 | 2 | 6 |
| NYHA class | | | |
| Unknown | 1 (<0.1%) | 0 (0%) | 1 (<0.1%) |
| NYHA CLASS II | 4,146 (69%) | 2,065 (69%) | 2,081 (69%) |
| NYHA CLASS III | 1,813 (30%) | 910 (30%) | 903 (30%) |
| NYHA CLASS IV | 41 (0.7%) | 23 (0.8%) | 18 (0.6%) |
| History of LVEF <40% | 273 (4.5%) | 126 (4.2%) | 147 (4.9%) |
| SGLT-2 inhibitor use at baseline | 817 (14%) | 424 (14%) | 393 (13%) |
| Time since index HF event 1 | | | |
| randomized during/at HF event | 749 (12%) | 360 (12%) | 389 (13%) |
| very recent (<= 7 days from randomization) | 470 (7.8%) | 250 (8.3%) | 220 (7.3%) |
| recent (>7 days - <= 3 months) | 2,028 (34%) | 998 (33%) | 1,030 (34%) |
| >3 months | 937 (16%) | 489 (16%) | 448 (15%) |
| no index HF event | 1,817 (30%) | 901 (30%) | 916 (31%) |

[1]n (%); Median (IQR)

*Supplementary Table 2: Baseline characteristics*

| Outcome | Finerenone, E (n;%), N=3003 (100%) | Placebo, E (n;%), N=2998 (100%) | Frequentist effect estimate (HR, RR, OR, diff. in means), p =p-value | Bayesian effect estimate (vague priors) |
|---|---|---|---|---|
| Event-of-interest: Cardiovascular Death and Total (First and Recurrent) Heart Failure event | 1083 (624, 20.8%) | 1283 (719, 24.0%) | 0.84 (0.74 to 0.95), p =0.007 | 0.83 (0.74-0.94), P(RR<1) = 99.85% |
| Worsening heart failure Events | 842 (479, 16.0%) | 1024 (573, 19.1%) | 0.82 (0.71 to 0.94), p =0.006 | 0.82 (0.73-0.93), P(RR<1) = 99.96% |
| Cardiovascular Death | 242 (242, 8.1%) | 260 (260, 8.7%) | 0.93 (0.78 to 1.11), p =0.412 | 0.93 (0.79-1.10), P(HR<1) = 80% |
| | | | | |
| First event-of-interest: Heart failure event and CV death | 624 (624, 20.8%) | 719 (719, 24.0%) | 0.84 (0.76 to 0.94), p =0.002* | 0.85(0.76-0.94), P(HR<1) = 99.88% |
| Improvement in NYHA at 12 months | 557/3003 (18.5%) | 553/2998 (18.4%) | 1.01 (0.88 to 1.15), p =0.930* | 1.01 (0.88-1.14), P(OR<1) = 46% |
| Change in KCCQ TSS between baseline and 12 months | 8.00 ± 0.32 | 6.42 ± 0.32 | 1.6 (0.79 to 2.3), p <0.001* | 1.58 (0.80-2.36), P(diff>0) > 99.99% |
| Composite renal endpoint | 75 (75, 2.5%) | 55 (55, 1.8%) | 1.33 (0.94 to 1.89), p =0.107* | 1.31 (0.94-1.82), P(HR<1) = 6% |
| All-cause mortality | 491 (491, 16.4%) | 522 (522, 17.4%) | 0.93 (0.83 to 1.06), p =0.279* | 0.94 (0.83-1.06), P(HR<1) = 85% |

*Supplementary Table 3: Frequentist and Bayesian (vague prior) estimates of treatment efficacy for all primary and secondary outcomes (* under strict alpha-spending rules and the hierarchical testing strategy these p-values should be disregarded but are presented here for completeness).*

| Prior and prior probability of treatment benefit | | RR; 95% Confidence Interval (CI)/ Credible Interval (CrI) of the posterior distribution | Posterior probability of treatment benefit greater than specific thresholds | | | |
|---|---|---|---|---|---|---|
| | | | Any benefit Pr(RR<1.0) | 10% reduction Pr(RR<0.9) | 15% reduction Pr(RR<0.85) | 20% reduction Pr(RR<0.8) |
| Bayesian posterior results with priors at original variance | | | | | | |
| Vague | 50% | 0.83 (95% CrI: 0.74-0.94) | 99.85% | 90% | 64% | 27% |
| TOPCAT | 97% | 0.83 (95% CrI: 0.75-0.92) | 99.98% | 93% | 64% | 22% |
| TOPCAT-Americas) | 99% | 0.82 (95% CrI: 0.74-0.91) | 99.99% | 96% | 74% | 32% |
| FIDELITY | 99.75% | 0.83 (95% CrI: 0.75-0.90) | >99.99% | 97% | 73% | 24% |

*Supplementary Table 4: Re-estimation of the primary treatment effect under various vague priors, with the variance of the prior set to the original value (not downweighted by doubling the variance as in the primary results. Summary of frequentist and Bayesian posterior estimates of the primary treatment effect of finerenone versus placebo. MAP=meta-analytic prior using data from TOPCAT-Americas and FIDELITY combined.* $Pr(RR < x)$ *shows the posterior probability that the RR is below critical value* $x$.

| | Placebo | Finerenone | Difference in the probability of experiencing the event (95% CrI) | Odds ratio (95% CrI) |
|---|---|---|---|---|
| Any SAE | 1378/2993 (46.0%) | 1359/2993 (45.4%) | -0.5 (-2.9, 1.9) | 1.0 (0.9, 1.1) |
| Any hyperkalemia (investigator reported) | 136/2993 (4.5%) | 311/2993 (10.4%) | 5.5 (4.2, 6.8) | 2.4 (1.9, 3.0) |
| Potassium > 5.5 mmol/L | 207/2915 (7.1%) | 426/2921 (14.6%) | 7.1 (5.6, 8.7) | 2.2 (1.9, 2.7) |
| Potassium > 6 mmol/L | 44/2915 (1.5%) | 90/2921 (3.1%) | 1.2 (0.6, 1.9) | 2.0 (1.4, 2.9) |
| Potassium < 3.5 mmol/L | 299/2915 (10.3%) | 145/2921 (5.0%) | -4.9 (-6.2, -3.6) | 0.5 (0.4, 0.6) |
| Creatinine ≥ 2.5 mg/dL | 110/2921 (3.8%) | 167/2928 (5.7%) | 1.8 (0.7, 2.8) | 1.5 (1.2, 2.0) |
| Creatinine ≥ 3 mg/dL | 45/2921 (1.5%) | 77/2928 (2.6%) | 0.9 (0.3, 1.6) | 1.7 (1.2, 2.4) |
| SBP < 90 mmHg | 95/2935 (3.2%) | 146/2934 (5.0%) | 0.9 (0.4, 1.6) | 1.6 (1.2, 2.0) |
| SBP < 100 mmHg | 374/2935 (12.7%) | 556/2934 (19.0%) | 5.6 (4.0, 7.1) | 1.7 (1.4, 1.9) |

*Supplementary Table 5: Safety outcomes on absolute and relative scales. Summaries of Bayesian posterior distributions using vague priors*
